# Molecular mechanism of infectious spleen and kidney necrosis virus in manipulating the hypoxia-inducible factor pathway to augment virus replication

**DOI:** 10.1080/21505594.2024.2349027

**Published:** 2024-04-29

**Authors:** Jian He, Yang Yu, Wenhui Liu, Zhimin Li, Zhang Qi, Shaoping Weng, Changjun Guo, Jianguo He

**Affiliations:** a State Key Laboratory for Biocontrol/Southern Laboratory of Ocean Science and Engineering (Guangdong, Zhuhai), Guangdong Provincial Observation and Research Station for Marine Ranching of the Lingdingyang Bay, School of Marine Sciences, Sun Yat-sen University, Guangzhou, PR China; b Guangdong Province Key Laboratory of Aquatic Economic Animals, Sun Yat-sen University, Guangzhou, PR China

**Keywords:** HIF-pathway, FIH, VHL, hypoxia, iridovirus

## Abstract

Infectious spleen and kidney necrosis virus (ISKNV), a member of the genus *Megalocytivirus* in the family *Iridoviridae*, can infect over 50 fish species and cause significant economic losses in Asia. Our previous study showed that hypoxia triggers the hypoxia-inducible factor pathway (HIF-pathway), leading to increased replication of ISKNV through promoting the upregulation of viral hypoxic response genes like *orf077r*. This study delved into the molecular mechanism of how ISKNV manipulates the HIF-pathway to enhance its replication. *In vitro* and *in vivo* experiments confirmed that ISKNV infection activated the HIF-pathway, which in turn promoted ISKNV replication. These findings suggest that ISKNV actively manipulates the HIF-pathway. Co-immunoprecipitation experiments revealed that the ISKNV-encoded protein VP077R interacts with the Von Hippel−Lindau (VHL) protein at the HIF-binding region, competitively inhibiting the interaction of HIF-1α with VHL. This prevents HIF degradation and activates the HIF-pathway. Furthermore, VP077R interacts with factor-inhibiting HIF (FIH), recruiting FIH and S-phase kinase-associated protein 1 (Skp1) to form an FIH – VP077R – Skp1 complex. This complex promotes FIH protein degradation via ubiquitination, further activating the HIF-pathway. These findings indicated that ISKNV takes over the HIF-pathway by releasing two “brakes” on this pathway (VHL and FIH) via VP077R, facilitating virus replication. We speculate that hypoxia initiates a positive feedback loop between ISKNV VP077R and the HIF pathway, leading to the outbreak of ISKNV disease. This work offers valuable insights into the complex interactions between the environment, host, and virus.

## Introduction

The hypoxia-inducible factor pathway (HIF-pathway) is one of the most critical oxygen-sensing regulatory networks that help organisms tolerate hypoxia [1]. The key regulator of this pathway is HIF-1α protein [2–4]. The enzyme prolyl hydroxylase domain-containing proteins (PHDs) and factor-inhibiting HIF (FIH) are critical regulators of HIF-1α [5]. Under normoxic conditions, PHDs hydroxylate two conserved proline residues in HIF-1α and regulate ubiquitin-dependent degradation of HIF-1α via Von Hippel−Lindau (VHL) [6]. FIH catalyzes asparagine hydroxylation of HIF-1α and blocks the association of HIF-1α with CBP/p300 transcriptional coactivators [7]. Under hypoxic conditions, PHDs and FIH are functionally inactive, leading to stabilization and accumulation of HIF-1α subunits. The stabilized HIF-1α subunits then translocate to the nucleus and dimerize with HIF-1β to form the active HIFs complex. The HIFs complex binds to hypoxia response elements (HREs) in the promoter regions of target genes (e.g. *glut-1*, *vegf*, and *ldha*), leading to increased transcription of genes involved in angiogenesis, erythropoiesis, metabolism, and other responses to hypoxia [8].

The HIF-pathway is an important target that various viruses manipulate [9]. Many viruses take advantage of this pathway to promote their own replication. For example, human cytomegalovirus infection influences the HIF-pathway by increasing HIF-1α mRNA levels [10]. Respiratory syncytial virus (RSV) infection in pulmonary epithelial cells leads to the stabilization of HIF-1α protein and subsequent transcription of HIF-1α target genes, creating an intracellular environment favorable for RSV replication [11]. Vaccinia virus activates HIF-pathway to benefit viral replication through viral protein C16, which binds to the PHD2 and inhibits PHD2-dependent hydroxylation of HIF-1α [12]. Human papillomavirus 16 E6 can hinder the interaction between HIF-1α and VHL, attenuating VHL-mediated HIF-1α ubiquitination and causing HIF-1α accumulation. Subsequently, the activated HIF-pathway induces the transcription of target genes to facilitate virus replication [13]. However, not all the viruses can take advantage of the activated HIF-pathway. For instance, the replication of Vesicular stomatitis virus is inhibited by activating the hypoxia-induced HIF-pathway [14]. The overexpression of HIF-1α can hinder the replication of H-1 Parvovirus [15]. However, whether viruses of lower vertebrates manipulate the HIF-pathway remain unknown.

The aquaculture industry provides over 17% of the world’s animal protein consumption, making it increasingly vital in meeting human food and nutritional needs [16]. However, the rapid expansion of aquaculture has led to an increase in disease occurrences, which have hindered its growth [16]. Infectious spleen and kidney necrosis virus (ISKNV), a member of the genus *Megalocytivirus* in the family *Iridoviridae*, can infect over 50 species of fish and cause significant economic losses in Asia aquaculture [17]. Our previous research found that hypoxic aquatic environments can promote the HIF-pathway, enhancing ISKNV replication through viral HREs in its genome [18]. However, it remains unclear whether ISKNV can actively manipulate the HIF-pathway. Therefore, the present study aimed to investigate the molecular mechanisms of ISKNV’s interaction with the HIF-pathway. Our findings could provide insights into how hypoxia triggers fish iridovirus disease outbreaks and contribute to the prevention and control of aquatic iridovirus diseases.

## Materials and methods

### Fish, cells, and virus

The mandarin fish (*Siniperca chuatsi*) used in this study, with a body weighed 50 ± 5 g were purchased from a farm in Guangdong province, China. The fish were kept indoor glass tanks, each with an air-pumped circulating water systems. The breeding water temperature was kept at 27 ± 1°C. The mandarin fish fry (MFF-1) cells were grown in Dulbecco’s modified Eagle medium (DMEM; GIBCO, USA) containing 10% foetal bovine serum (FBS; Hyclone, USA) at 27°C under a humidified atmosphere with 5% CO_2_ [19]. The ISKNV strain (NH-2017) was isolated in 2017 [20]. For virus infections, cells were seeded in plates for 24 h and then exposed to ISKNV at a concentration of 1 µL with a dilution of 1.96 × 10^9^ TCID_50_/mL in 1 mL of culture medium. Mandarin fish samples were injected intraperitoneally with 100 µL of ISKNV at a concentration of 1.96 × 10^8^ TCID_50_/mL.

### Reagents and antibodies

The reagents and antibodies used in this study were listed in Table 1.

**Table 1.** The reagents and antibodies used in this study.

REAGENT or RESOURCE	SOURCE	IDENTIFIER
**Antibodies**		
Anti-ha	CST	3724S
Anti-myc	Sigma-Aldrich	M4439
Anti-flag	Sigma-Aldrich	F7425
Anti-his	Proteintech	66005–1-Ig
Anit-GFP	Proteintech	50430–2-AP
Anti-HIF-1α	Abcam	ab179483
Anti-ISKNV VP101R	Unpublished data by Dr. Dong CF	mAb2D8
Anti-Histone H3	CST	5192
Anti-β-actin	Proteintech	66009–1
Anti-Rabbit Ig HRP Conjugate	promega	W401B
Anti-Mouse Ig HRP Conjugate	Promega	W4021
**Virus Strains**		
ISKNV	This study	
**Chemicals, Peptides, and Recombinant Proteins**		
DMSO	MP	196055
DMOG	MCE	HY-15893
BAY87–2243	MCE	HY-15836
MG132	Sigma-Aldrich	M8699-1 MG
CHX	MCE	HY-12320
**Critical Commercial Assays**		
Dual Luciferase Reporter Gene Assay Kit	Promega	Cat# E2920
DNeasy Blood & Tissue Kits	Qiagen	Cat# 69504
RNeasy Mini Kit	Qiagen	Cat# 74104
**Experimental Models: Cell Lines**		
MFF-1 cell lines	This study	Ref. 8
**Experimental Models: Organisms/Strains**		
Mandarin fish	This study	
**Oligonucleotides**		
Primers for cloning and qRT-PCR: Table 2	This study

**Table 2.** Primers used in this study.

Primer	Sequence (5’-3’)
pCMV-myc-*orf077r* (F)	GGAATTCGGATGGATTCCCTTGTCGACCT
pCMV-myc-*orf077r* (R)	GGGGTACCTAGTGTTTTATATACCCCAAAGTCAATGTGT
pEGFP-N3-*orf077r* (F)	GGAATTCTATGGATTCCCTTGTCGACCT
pEGFP-N3-*orf*077*r* (R)	GGGGTACCTAGTGTTTTATATACCCCAAAG
pCMV-myc-*orf077r*-ΔN (F)	GGAATTCGGGATGAGTACAATGCGGAG
pCMV-myc-*orf077r*-ΔN (R)	GGGGTACCTCATAGTGTTTTATATACCCCAAAG
pCMV-myc-*orf077r*-ΔC (F)	GGAATTCGGGATTCCCTTGTCGACCTCATCCGGGC
pCMV-myc-*orf077r*-ΔC (R)	GGGGTACCTCATGCCACGTCGTGTTTGTCATC
Overlap-*orf077r*-ΔANK1 (F)	GGAATTCGGATGGATTCCCTTGTCGAC
Overlap-*orf077r*-ΔANK1 (R)	GGGGTACCTCATAGTGTTTTATATACCCCAAAG
Overlap-*orf077r*-ΔANK2 (F)	GGAATTCGGATGGATTCCCTTGTCGAC
Overlap-*orf077r*-ΔANK2 (R)	GGGGTACCTCATAGTGTTTTATATACCCCAAAG
Overlap-*orf077r*-ΔANK3 (F)	GGAATTCGGATGGATTCCCTTGTCGAC
Overlap-*orf077r*-ΔANK3 (R)	GGGGTACCTCATAGTGTTTTATATACCCCAAAG
Overlap-*orf077r*-ΔANKs (F)	GGAATTCGGATGGATTCCCTTGTCGAC
Overlap-*orf077r*-ΔANKs (F)	GGGGTACCTCATAGTGTTTTATATACCCCAAAG
pCMV-myc-GFP (F)	GGAATTCGGATGGTGAGCAAG
pCMV-myc-GFP (R)	GGGGTACCTTACTTGTACAGCTCGT
pCMV-ha-FIH (F)	GGAATTCGGGAAGCGCCGACCGTTGTAGAG
pCMV-ha-FIH (R)	GGGGTACCCTAACTGTGATCCTGGTCATATCG
pCMV-ha-FIH-ΔN (F)	GGAATTCGGTATATCTGCAGCAGACCTTGAATGACAC
pCMV-ha-FIH-ΔN (R)	GGGGTACCCTAACTGTGATCCTGGTCATATCGTCCCT
pCMV-ha-FIH-ΔC (F)	GGAATTCGGGAAGCGCCGACCGTTGTAGAGGCTGACC
pCMV-ha-FIH-ΔC (R)	GGGGTACCTCGCAGGGGGTATTCTATCCTCTTGGGT
Overlap-FIH-ΔJmjc (F)	GGAATTCGGGAAGCGCCGACCGTTGTAGAG
Overlap-FIH-ΔJmjc (R)	GGGGTACCCTAACTGTGATCCTGGTCATATCG
pCMV-ha-VHL (F)	GGAATTCGG CCTCAAGAGGGAGAGCAGTCT
pCMV-ha-VHL (R)	GGGGTACC TCATTCCTCCTGGCTTTGAATGCTC
Overlap-VHL-ΔHIF-binding site (F)	CCATCGTGTGTCCCTGCATCCCGCGGCCGCCCGGCCTCCA
Overlap-VHLΔHIF-binding site (R)	AGATGGAGGCCGGGCGGCCGCGGGATGCAGGGACACACGATGGGCTTCTGGT
pCMV-flag-HIF-1α (F)	GGAATTCGG GACACAGGAATTGTACCAGAAAAG
pCMV-flag-HIF-1α (R)	GGGGTACCTCAGTTGACGTGGTCCAGAGC
pEGFP-N3-HIF-1α (F)	GGAATTCTATGGACACAGGAATTGTACCAGAAAAG
pEGFP-N3-HIF-1α (R)	GGGGTACCGTTGACGTGGTCCAGAGC
pCMV-flag-Skp1 (F)	GAAGATCTTCCCCACAATAAAATTACAGAGCTCTGATG
pCMV-flag-Skp1 (R)	AGATCTGCGGCCGCTAAACTATTTACTTTTCTTCACACCACTGGTTCTCT
RT-qPCR-*orf077r* (F)	GAGTACAATGCGGAGGGCTTCA
RT-qPCR-*orf077r* (R)	TGGCCATCGTTGGGGTCTG
RT-qPCR-*mcp* (F)	CAATGTAGCACCCGCACTGACC
RT-qPCR-*mcp* (R)	ACCTCACGCTCCTCACTTGTC
RT-qPCR-*vegf* (F)	AGAAAGCGTTTGTTCGTGC
RT-qPCR-*vegf* (R)	TCTTGCTGGCGTTCTTCAC
RT-qPCR-*glut1* (F)	GGTTTATTGTGGCAGAGTTGTT
RT-qPCR-*glut1* (R)	CCCACTATGAAGTTGGCAGTC
RT-qPCR-*ldha* (F)	GGTCTTCCTGAGCATCCCTT
RT-qPCR-*ldha* (R)	TTCTCCTCTTCGGGCTTCA
RT-qPCR-*β-actin* (F)	CCCTCTGAACCCCAAAGCCA
RT-qPCR-*β-actin* (R)	CAGCCTGGATGGCAACGTACA
qPCR-*mcp* (F)	CAATGTAGCACCCGCACTGACC
qPCR-*mcp* (R)	ACCTCACGCTCCTCACTTGTC

### Dual luciferase reporter assays

To determine whether ISKNV infection can induce the HIF-pathway. The relative luciferase activity (RLA) of HRE-luc was used to verify in this study. MFF-1 cells were cultured in 24-well plates for 24 h, then co-transfected with the HRE-luciferase reporter plasmid (pGL4-HREs-luc), each tested plasmid, and pRL-TK plasmid. The pRL-TK plasmid was transfected as an internal control. After 2 h, the transfection mixture was replaced with 500 µL of DMEM. Then, infected with ISKNV at 6 h post-transfection. After48 h post-infection (p.i.), the total cell lysates were subjected to Dual Luciferase Reporter Gene Assay Kit (Promega, USA) according to the manufacturer’s instructions. Luciferase activities were measured using Glomax (Promega, USA). All experiments were performed in at least three independent experiments with three technical replicates for each experiment.

### Mandarin fish and MFF-1 cells treated with DMOG and BAY87–2243

To investigate whether HIF-pathway activation benefits ISKNV replication, we employed specific activator (DMOG, Dimethyloxallyl Glycine) and inhibitor (BAY87–2243, 1-Cyclopropyl-4-{4-[(5-methyl-3-{3-[4-(trifluoromethoxy)phenyl]-1,2,4-oxadiazol-5-yl}-1 H-pyrazol-1-yl)methyl]pyri-din-2-yl}piperazine) of HIF-pathway [21]. DMOG was prepared as a storage solution in DMSO (1 M). BAY87–2243 was prepared as a storage solution in DMSO (10 mM). MFF-1 cells were treated with DMOG (1 mM) or BAY87–2243 (100 nM) at 4 h after ISKNV infection [18]. DMOG and BAY87–2243 were prepared as a storage solution (2.5 mg/mL) according to the following protocol: 100 μL of a 25.0 mg/mL DMSO stock solution was added to 400 μL of PEG300, mixed well. Then, 50 μL of Tween-80 was added to the mixture, and it was mixed well again. Finally, 450 μL of normal saline was added to make up a total volume of 1 mL. Fish were treated with DMOG or BAY 87–2243 at a concentration of 1 μg/g by intraperitoneal injected [22,23].

### Gene expression quantification by quantitative reverse transcription PCR (RT-qPCR)

Total RNAs were isolated from MFF-1 cells (including normal cells, cells exposed to viruses and cells transfected with plasmid) and fishes (including normal fish, fish injected with ISKNV, and fish treated with DMOG or BAY87–2243). All total RNA samples were extracted using the RNeasy mini kit (Qiagen, Germany) and then reverse transcribed to cDNA using the PrimeScript RT Reagent kit (TaKaRa, China) according to the manufacturer’s instructions. RT-qPCR assays were performed on a LightCycler480 instrument. The primers used in this study are listed in Table 2. The PCR reaction mixture (10 μL) contained 5 μL 2× SYBR Premix Ex Taq (TaKaRa, China), 1 μL of DNA template, 0.2 μL of 10 μM primers, and 3.6 μL of H_2_O. The absolute qPCR conditions were as follows: one cycle at 95°C for 10 s, followed by 40 cycles of 5 s at 95°C, 40 s at 60°C, and 1 s at 72°C. RT-qPCR was performed in triplicate for each sample.

### Absolute quantitative real-time PCR (qPCR)

The amount of viral major capsid protein (*isknv-mcp*) gene copies was used to determine the level of ISKNV genomic copies. The number of *isknv-mcp* gene copies was determined using the standard curve method of qPCR using a LightCycler480 instrument (Roche Applied Science, Germany), as previously described [24]. The primers used in this study were listed in Table 2. Absolute qPCR was performed in triplicate for each sample.

### TCID_50_ assay

Viral titres were also used to determine ISKNV replication under different treatments (MFF-1 cellls were treated with DMOG or BAY87–2243). Before infection, 96-well dishes were seeded with cells. Dilutions of the original virus sample were made in culture medium, ranging from 10^−1^ to 10^−10^. 0.1 mL of virus dilution was added to 10 wells for each dilution, while 0.1 mL of culture medium served as a negative control. The dishes were incubated at 27°C. The number of positive and negative wells was recorded. The TCID_50_ was calculated using the Karber method in MFF-1 cells [25].

### Co-immunoprecipitation (Co-IP)

Co-IP assays were used to identify protein-protein interactions in this study. Cells were grown in 75 cm^2^ flasks and transfected with plasmids. At 24 h post-transfection, cells were lysed with ice-cold lysis buffer containing 10 mM Tris-HCl pH 7.5, 0.4 M NaCl, 1% NP-40, 0.4% Triton X-100, 0.2% sodium deoxycholate, 1 mM EDTA, and protease inhibitors (Calbiochem, USA) for 30 min. Cellular debris was removed by centrifugation at 12,000 g for 15 min at 4°C. The lysates were immunoprecipitated with corresponding antibodies in a rotation wheel for 2 h and subsequently adsorbed onto Pierce™ Protein A/G Magnetic Beads (Rockford, USA) in a rotation wheel for 1 h. The beads were washed five times with 1 mL of wash buffer (10 mM Tris-HCl, pH 7.5, 0.2 M NaCl, and 1 mM EDTA) on a roller for 10 min each time, followed by centrifugation at 2,000 g at 4°C for 3 min. Final immunoprecipitates and the whole cell lysates were resuspended in SDS loading buffer and analysed by immunoblotting (IB) using indicated antibodies.

### Western blotting

The protein samples were mixed with 5× loading buffer (250 mM Tris-HCl pH 6.8, 10% SDS, 5% β-mercaptoethanol, 50% glycerinum, and 0.5% bromophenol blue), boiled for 10 min, and then subjected to SDS-PAGE for separation. The separated proteins were subsequently transferred onto nitrocellulose membranes (Amersham Biosciences, USA). The nitrocellulose membranes were blocked in blocking buffer [5% (w/v) skim milk dissolved in TBST buffer (20 mM Tris-HCl, 150 mM NaCl, 0.1% Tween 20, and pH 7.6)] at room temperature for 1 h. The membranes were washed three times for 10 min each with TBST buffer. After blocking, the membranes were incubated with the antibody of interest (Table 1, Key resources table) at room temperature for 2 h. The membranes were then washed three times for 10 min each with TBST buffer. In contrast, some membranes were incubated with goat anti-mouse/rabbit IgG HRP conjugate (Promega, USA) at room temperature for 1 h. The membranes were then washed three times for 10 min each with TBST buffer. Protein bands were visualized using a high-sig chemiluminescence WB substrate kit (Tanon, China).

### Quantification and statistical analysis

All analyses were performed using SAS v9.3. For all analyses, significance was set at the 0.05 threshold (NS, not significant; *p < 0.05; **p < 0.01).

## Results

### The ISKNV infection activates the HIF-pathway

**Figure 1.**
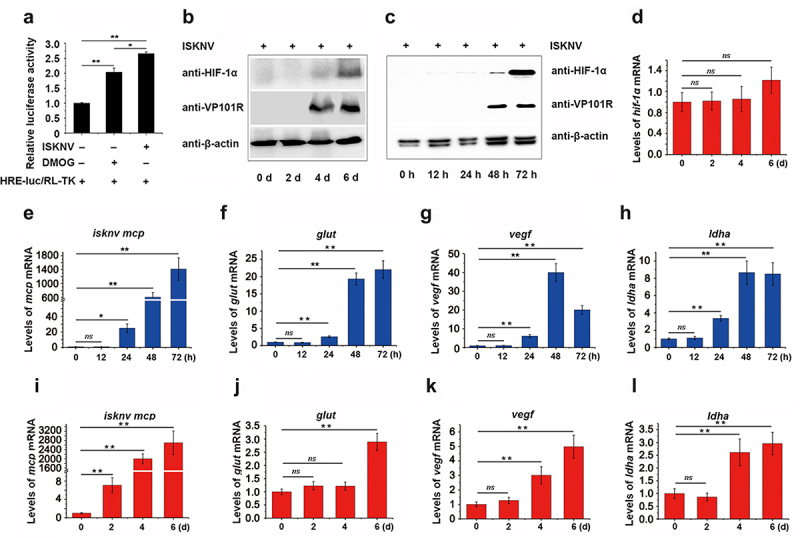
HIF-pathway is activated by ISKNV infection. (a) Cells were co-transfected with pGL4-HREs-luc and pRT-TK plasmids and then infected with ISKNV at 6 h post-transfection. The activation of the HIF-pathway was detected using a dual-reporter assay at 48 h post-infection (p.I.). (b,i–l) Spleen samples were collected from infected mandarin fish at 0-, 2-, 4-, and 6-day p.I. (c,e–h) ISKNV-infected cells were collected at 12-, 24-, 48-, and 72-h p.I. Anti-HIF-1α and anti-β-actin (as an internal control) were used in western blot experiments, and the relative expression levels of *isknv mcp*, *glut-1, vegf*, and *ldha* were determined using RT-qPCR. (d) Expression levels of *hif-1α* after ISKNV infection. Spleen samples were collected from infected mandarin fish at 0-, 2-, 4-, and 6-days p.I. The relative expression levels of *hif-1α*, *glut-1*, *vegf*, and *ldha* were detected using RT-qPCR.

To determine whether ISKNV can induce the HIF-pathway under normoxic conditions, we examined the activation of the HIF-pathway and the transcription level of HIF-1α in both fish and cellsinfected with ISKNV. As shown in Figure 1(a), the relative luciferase activity (RLA) of HRE-luc significantly increased at 48 h post-infection with ISKNV in cells. The protein levels of HIF-1α also increased significantly at 4–6-day post-infection in fish (Figure 1(b)) and at 48–72 h post-infection in cells (Figure 1(c)). Additionally, the expression levels of the downstream genes of the HIF-pathway (*glut-1*, *vegf*, and *ldha*) gradually increased in cells (Figures. 1(e–h)) and fish (Figure 1(i–l)) infected with ISKNV. Notably, the transcription level of HIF-1α did not change significantly during the 0–6 days post-infected in fish (Figure 1(d)). These observations suggested that ISKNV infection strongly promotes the activation of the HIF-pathway both *in vivo* and *in vitro*, indicating that ISKNV may manipulate the HIF-pathway.

### The ISKNV protein VP077R promotes the activation of HIF-pathway

**Figure 2.**
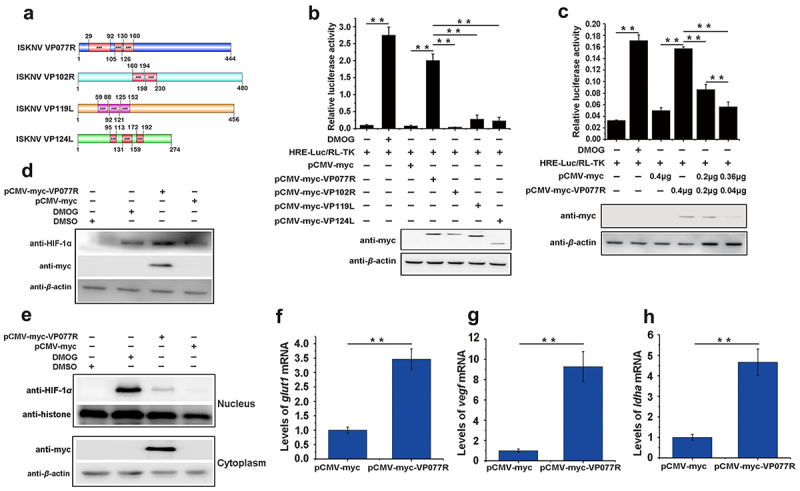
The ISKNV VP077R promotes the HIF-pathway. (a) Analysis of the ankyrin-repeat-containing domain of ISKNV VP077R, VP102R, VP119L, and VP124L. (b–c) The activation of the HIF-pathway was detected using dual-reporter assay at 48 h post transfection. (d–e) The protein levels of HIF-1α in cells were measured after overexpression of pCMV-myc-VP077R or pCMV-myc (control) for 24 h. Cells were treated with DMSO or DMOG before collection and extraction of nuclei. HIF-1α and histone (as an internal control for nuclear protein) were detected using anti-HIF-1α and anti-histone. VP077R and β-actin (as an internal control) were detected using anti-myc and anti-β-actin antibodies. (f–h) The relative expression levels of *glut-1*, *vegf*, and *ldha* were measured in MFF-1 cells transfected with pCMV-myc-VP077R or pCMV-myc (control).

Multiple ankyrin repeat (ANK) proteins have been shown to regulate the HIF-pathway [26,27]. To explore whether ANK proteins encodes by ISKNV are involved in modulating the HIF-pathway, we analysed four ISKNV viral ANK proteins: VP077R, VP102R, VP119L, and VP124L (Figure 2(a)). We then preliminarily screened their roles in the HIF-pathway. As shown in Figure 2(b), overexpression of VP077R led to a significant increase in the RLA of HRE-luc, while overexpression of VP102R, VP119L, or VP124L did not product any changes. Further investigation confirmed the effect of VP077R on the HIF-pathway. Specifically, VP077R overexpression increased the RLA of HRE-luc in a dose-dependent manner (Figure 2(c)), elevated the protein level of endogenous HIF-1α (Figure 2(d)), and significantly increased the amount of HIF-1α entering the nucleus compared to control cells (Figure 2(e)). Moreover, overexpressed VP077R significantly induced the transcription levels of the downstream genes of the HIF-pathway, including *glut-1*, *vegf*, and *ldha* (Figure 2(f–h)) (*p <*0.01). These results indicated that ISKNV VP077R promoted the activation of HIF-pathway.

### The ISKNV protein VP077R interacts with VHL and competitively inhibits its interaction with HIF-1α

**Figure 3.**
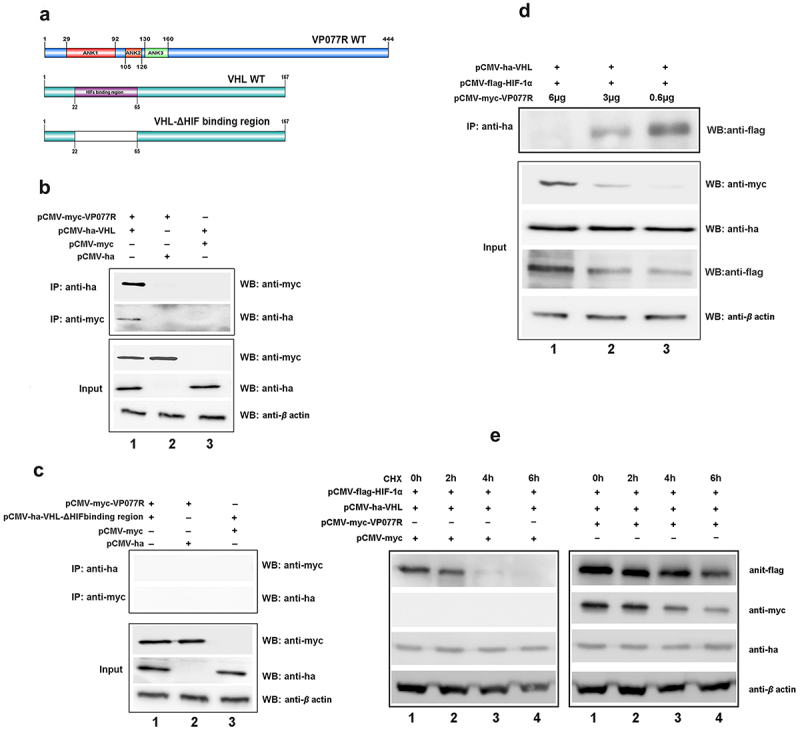
The ISKNV VP077R interacts with VHL and competitively inhibits the interaction between HIF-1α and VHL. (a) Description of VP077R, VHL, and various mutants used in this study. (b) The interaction between VP077R with VHL was detected using CO-IP assays. Cells were co-expressed with myc-VP077R/ha-VHL, myc-VP077R/ha-tag, and myc-tag/ha-VHL. Immunoprecipitation of myc-VP077R was performed using anti-myc antibody, and immunoprecipitation of ha-VHL was performed using anti-ha antibody. (c) Cells were co-expressed with the indicated plasmids. Immunoprecipitation of myc-VP077R was performed using anti-myc antibody, and while immunoprecipitation of the ha-VHL-ΔHIF binding region was performed using anti-ha. (d) Cells were expressed with 6 μg, 3 μg, or 0.6 μg of myc-VP077R, as well as co-expressed with ha-VHL, and flag-HIF-1α. Immunoprecipitation of ha-VHL was performed using anti-ha and detection of flag-HIF-1α was performed using anti-flag. (e) Cells were treated with CHX at 24 h after transfection with the indicated plasmid and then were collected and detected at 0 h, 2 h, 4 h, 6 h, using the anti-flag, anti-myc, anti-ha and anti-β-actin antibodies.

To explore the mechanism of VP077R regulation of the HIF-pathway, we identified the interaction between VP077R and VHL using Co-IP assay. As shown in Figure 3(b), myc-tagged VP077R was able to co-precipitate with ha-tagged VHL, and vice versa, indicating that VP077R physically interacts with VHL. VHL is the primary E3 ubiquitin ligase responsible for HIF-1α ubiquitin degradation via the ubiquitin-dependent pathway. Disturbing the interaction between VHL and HIF-1α can effectively activate the HIF-pathway. To investigate whether VP077R can interfere with the interaction between VHL and HIF-1α, Co-IP assays were performed in cells co-expressing ha-tagged VHL, flag-tagged HIF-1α, and different doses of myc-tagged VP077R. As shown in Figure 3(d), overexpression of VP077R disturbed the amount of the HIF-1α that was co-precipitated by VHL in a dose-dependent manner. Overexpression of VP077R strongly inhibited the degradation of HIF-1α by VHL (Figure 3(e)). These results indicate that VP077R can inhibit the interaction between HIF-1α with VHL, enhancing the stability of HIF-1α. Furthermore, we identified the binding sites of VP077R on VHL. Mutant domains of VHL (VHL-ΔHIF binding region) were constructed (Figure 3(a)). As shown in Figure 3(c), myc-tagged VP077R was unable to co-precipitate with ha-tagged VHL-ΔHIF binding region, and vice versa, indicating that the HIF binding region of VHL may be the main interaction site for VP077R. These results suggested that VP077R competitively binds to the VHL-HIF binding region, leading to suppression of HIF-1α degradation.

### The ISKNV protein VP077R interacts with FIH and promotes the degradation of FIH protein via ubiquitination

**Figure 4.**
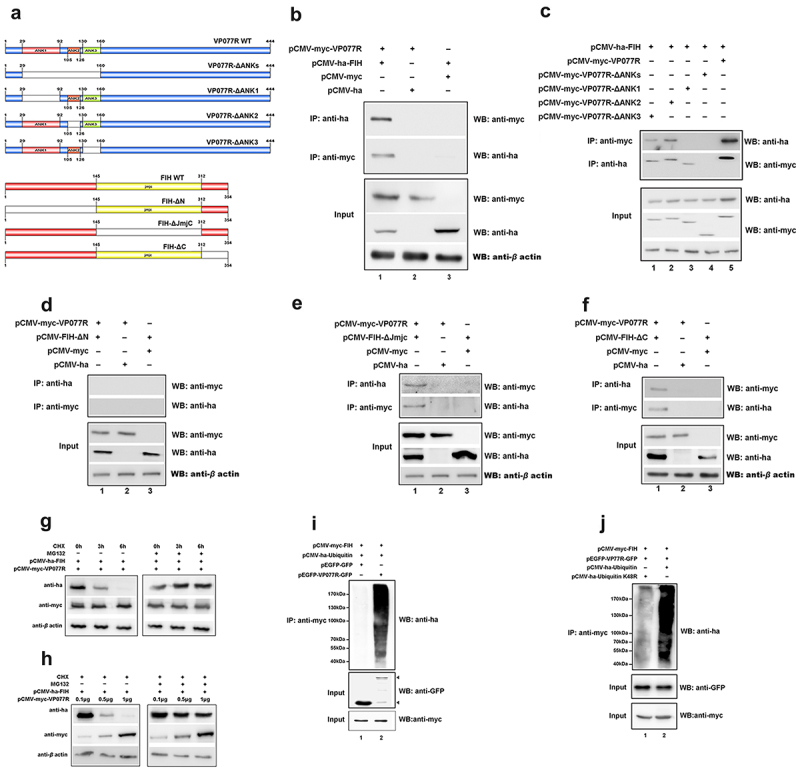
ISKNV VP077R interacts with FIH and promots the degradation of FIH protein by ubiquitin. (a) Description of VP077R, FIH, and various mutants used in this study. (b) The interaction between VP077R and FIH was determined using CO-IP assays. Cells were co-expressed with myc-VP077R/ha-FIH, myc-VP077R/ha-tag, and myc-tag/ha-FIH. Immunoprecipitation of myc-VP077R was performed using anti-myc and immunoprecipitation of ha-FIH was performed using the anti-ha. Used anti-myc, anti-ha, anti-β-actin to detect VP077R, FIH, and β-actin (as an internal control). (c) Cells were co-expressed with ha-FIH and myc-tagged VP077R or its mutants. Immunoprecipitation of myc-tagged VP077R or its mutants was performed using anti-myc, and in turn immunoprecipitation of the ha-FIH protein was performed using anti-ha. (d–f) Cells were co-expressed with myc-VP077R/ha-tagged FIH mutants (lane 1), myc-VP077R/ha-tag (lane 2), and ha-tagged FIH mutants/myc-tag (lane 3). Immunoprecipitation of myc-VP077R was performed using anti-myc, and immunoprecipitation of ha-tagged FIH mutants was performed using anti-ha. (g–h) Cells were treated with CHX and MG132 (right panel)/DMSO (left panel) at 24 h post-transfection with pCMV-myc-VP077R and pCMV-ha-FIH, and then the cells were collected at indicated times. (i) Cells were co-expressed with myc-FIH, ha-ubiquitin, and GFP (lane 1) or GFP-VP077R (lane 2). Immunoprecipitation of myc-FIH was performed using anti-myc and detection of poly-ubiquitin chains was performed using anti-ha. (j) Cells were co-expressed with GFP-VP077R, myc-FIH, and ha-ubiquitin or ha-ubiquitin K48R. Immunoprecipitation of myc-FIH was performed using anti-myc and detection of poly-ubiquitin chains was performed using anti-ha antibody.

FIH is another key protein that negatively regulates the HIF-pathway, so we also explored the effect of VP077R on FIH. As shown in Figure 4(b), myc-tagged VP077R was co-precipitated with ha-tagged FIH, and vice versa, indicating that VP077R physically interacts with FIH. To determine which regions of VP077R could bind to FIH, three-types of mutant domains—FIH (FIH-ΔN, -ΔC and -ΔJmjC domain) and four-types mutant domains of VP077R (VP077R-ΔANK-1, -ΔANK-2, -ΔANK-3, or –Δ all ANKs) were constructed (Figure 4(a)). Myc-tagged VP077R was able to co-precipitate ha-tagged FIH-ΔC (Figure 4(f)) and ha-tagged FIH-ΔJmjC-domain (Figure 4(e)), but not ha-tagged FIH-ΔN (Figure 4(d)), indicating that the N-terminal of FIH is the main binding region with VP077R. Furthermore, myc-tagged VP077R-ΔANK-2 and myc-tagged VP077R-ΔANK-3 were able to co-precipitate ha-tagged FIH, whereas myc-tagged VP077R-Δall-ANKs and myc-tagged VP077R-ΔANK-1 were not able to co-precipitate with ha-tagged FIH (Figure 4(c)). These results indicated that the ANKs domains of VP077R were the main binding region for FIH.

To further investigate the impact of VP077R on FIH, we measured the protein level of FIH in cells overexpressing VP077R. As shown in Figure 4(g), overexpression of VP077R significantly reduced the protein level of FIH. To determine whether VP077R reduces the FIH protein level via the proteasome pathway, we used MG132 to inhibit the ubiquitin proteasome system. As shown in Figure 4(h), MG132 impaired the effect of VP077R on decreasing the protein level of FIH. To further investigate how VP077R decreased the FIH protein level via the ubiquitin system, cells were co-transfected with myc-tagged FIH, ha-tagged ubiquitin, and VP077R-GFP tag (or with GFP as control). The results showed that myc-tagged FIH poly-ubiquitination was immunoprecipitated by the anti-ha antibody (Figure 4(i)), suggesting that VP077R could promote FIH degradation via ubiquitination. Furthermore, we identified that VP077R mediates K48-linked ubiquitination of FIH. Cells were co-overexpressed with myc-tagged FIH, ha-tagged ubiquitin (or ha-tagged ubiquitin K48R), and VP077R-GFP tag, and then the FIH poly-ubiquitination was determined via immunoprecipitation of myc-tagged FIH. The results showed that FIH poly-ubiquitination was significantly inhibited in cells were overexpressed with ha-ubiquitin K48R, compared to those overexpressed with ha-ubiquitin (Figure 4(j)). These observations suggested that VP077R promoted K48 poly-ubiquitination of FIH to decrease the protein levels of FIH.

### The FIH – VP077R – Skp1 complex facilitated the ubiquitination and degradation of FIH

**Figure 5.**
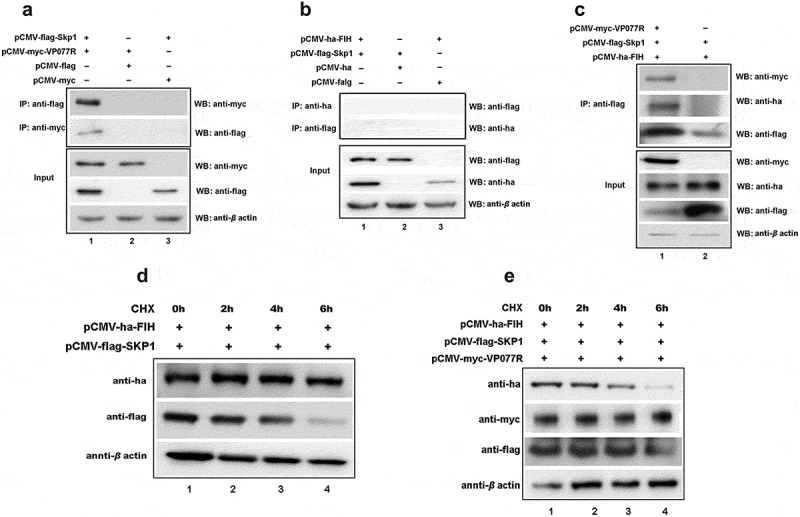
The “FIH – ISKNV VP077R – Skp1 complex” promotes FIH ubiquitination and degradation. (a) Cells were co-expressed with myc-VP077R/flag-Skp1, myc-VP077R/flag-tag, or myc-tag/flag-Skp1. Immunoprecipitation of myc-VP077R was performed using anti-myc, and immunoprecipitation of flag-Skp1 was performed using anti-flag. (b) Cells were co-expressed with ha-FIH/flag-Skp1, ha-tag/flag-Skp1, or ha-FIH/flag-tag. Immunoprecipitation of ha-FIH was performed using anti-ha, and in turn immunoprecipitation of flag-Skp1 was performed using anti-flag. (c) Cells were co-expressed with myc-VP077R or myc-tag, ha-FIH, and flag-Skp1. Immunoprecipitation of flag-tagged Skp1 protein was performed using anti-flag. (d–e) Cells were treated with CHX at 24 h after transfection with the indicated plasmid. Detections of VP077R, FIH, Skp1, and β-actin (as an internal control) were performed using anti-myc, anti-ha, anti-flag, and anti-β-actin, respectively.

Many viral ANK proteins engage cellular proteins through their ANK repeat motifs, targeting them for ubiquitin-mediated degradation within the SCF E3 complex [28]. We hypothesized that VP077R might promote FIH ubiquitination and degradation via SCF E3 complex. To identify the specific E3 ubiquitin ligase recruited by VP077R to mediate FIH ubiquitination, we investigated the interactions between S-phase kinase-associated protein 1 (Skp1), an adaptor protein of the SCF E3 complex [29], VP077R, and FIH using Co-IP assays. Myc-tagged VP077R could co-precipitate flag-tagged Skp1, and vice versa (Figure 5(a)), indicating an interaction between these two proteins. However, ha-tagged FIH failed to co-precipitate flag-tagged Skp1 (Figure 5(b)). To assess whether VP077R facilitated the assembly of an FIH – ISKNV VP077R – Skp1 complex, we conducted immunoprecipitation assays. Flag-tagged Skp1 could simultaneously co-precipitate both myc-tagged VP077R and ha-tagged FIH when cells were co-expressed with all three proteins (Figure 5(c)). These findings suggest that VP077R recruits both FIH and Skp1 to form the FIH – VP077R – Skp1 complex. Notably, overexpression of Skp1 alone did not enhance FIH degradation, whereas co-overexpression of VP077 and Skp1 significantly induced FIH degradation (Figure 5(e)). Taken together, these results indicated that VP077R recruited FIH and Skp1 to form the FIH – VP077R – Skp1 complex, promoting the FIH ubiquitination, degradation, and activation of the HIF-pathway.

### The activation of the HIF-pathway boosted ISKNV replication

**Figure 6.**
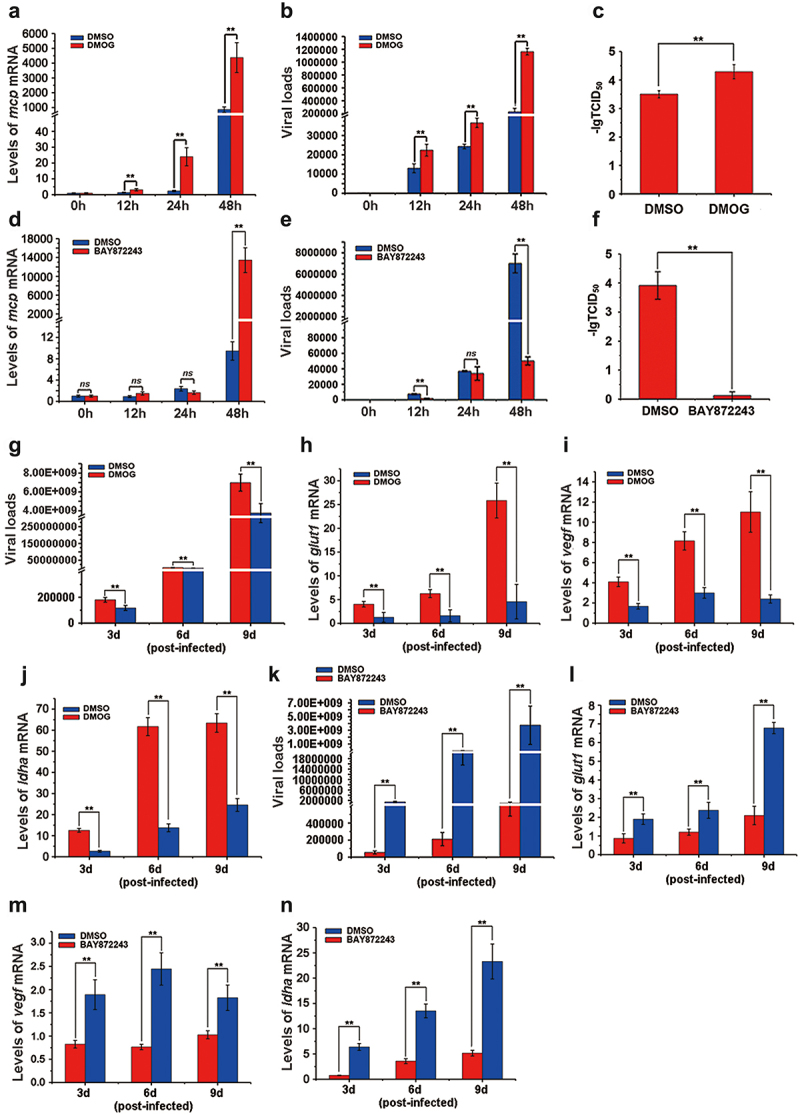
The activation of the HIF-pathway promotes ISKNV replication. (a–f) Cells were treated with DMOG (1 mM) or BAY87–2243 (100 nM) at 4 h after ISKNV infection, and then the levels of ISKNV infection were determined at indicated times. (a,d) levels of *isknv mcp* mRNA were detected using RT-qPCR. (b,e) Levels of viral genomic DNA were detected using absolute qPCR. (c,f) Viral titres (TCID_50_) of the cell lysates were determined by repeated freezing and thawing three times. (g–n) Fish were injected intraperitoneally with DMOG or BAY87–2243 at 4 h after ISKNV infection, and were given supplementary injections every three days. The spleen samples were separated and then the viral loads (g,k) and the transcription levels of the downstream genes (*glut-1*, *vegf*, and *ldha*) of the HIF-pathway (h–j, l–n) were determined.

To investigate whether HIF-pathway activation benefits ISKNV replication, we employed specific activator (DMOG) and inhibitor (BAY87–2243) of HIF-pathway. Cells were treated with DMOG or BAY87–2243 4 h post-ISKNV infection, and then the levels of ISKNV infection were determined at various time points. The levels of ISKNV *mcp* mRNA (Figure 6(a)), viral loads (Figure 6(b)), and viral titres (Figure 6(c)) were remarkably upregulated in the cells treated with DMOG, compared to those treated with DMSO (control group) (*p* < 0.01), while these parameters were significantly downregulated in cells treated with BAY87–2243 (Figures. 6(d–f)) (*p* < 0.01). Moreover, we explored the effects of DMOG and BAY87–2243 on ISKNV replication *in vivo*. Mandarin fish were injected intraperitoneally with DMOG or BAY87–2243 4 h post-ISKNV infection, with supplementary injections every three days. Spleen samples were collected and viral loads were determined. DMOG significantly promoted ISKNV replication (Figure 6(g)) and upregulated the transcription levels of the HIF-pathway downstream genes (*glut-1*, *vegf*, and *ldha*) (Figures. 6(h–j), *p <* 0.01), while BAY87–2243 significantly inhibited ISKNV replication (Figure 6(k)) and downregulated the transcription levels of these downstream genes (*glut-1*, *vegf*, and *ldha*) (Figures. 6(l–n), *p* < 0.01). These results suggested that the activation of the HIF-pathway promoted ISKNV replication.

## Discussion

**Figure 7.**
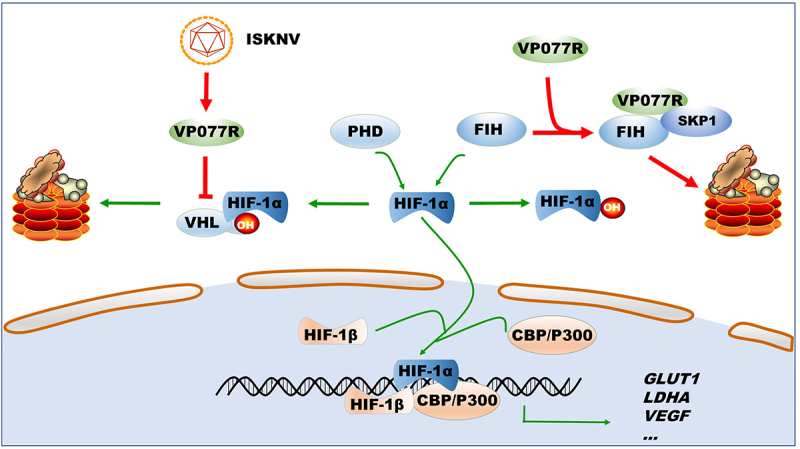
Model of how ISKNV protein VP077R regulates the HIF-pathway. ISKNV VP077R binds to VHL and competitively inhibits its interaction with HIF-1α, stabilizing the level of HIF-1α protein. Additionally, ISKNV VP077R interacts with FIH, promoting its ubiquitin-dependent degradation and maintaining the transcription activity of HIF-1α.

Under normoxic conditions, the stability of the HIF-pathway is strictly regulated by two key mechanisms in both mammals and lower vertebrates [5,30]. The first mechanism involves PHDs and VHL, which cause ubiquitin-dependent degradation of HIF-1α [31–33]. The second is FIH, which negatively regulates the association of HIF-1α with CBP/p300 transcriptional coactivators [34,35]. As such, PHDs-VHL and FIH are prime targets for viruses seeking to activate the HIF-pathway. For instance, the latent membrane protein 1 of EBV upregulates the seven in absentia homolog 1 (Siah1) E3 ubiquitin ligase, leading to proteasomal degradation of PHD1 and PHD3 [36]. Hepatitis B virus X protein inhibited VHL binding to HIF-1α, thereby preventing HIF-1α degradation [37]. However, reports on virus-targeted FIH regulation of the HIF-pathway are scarce. Influenza A virus infection can reduce FIH expression levels [38], while Orf virus (ORFV) sequestrates FIH to activate the HIF-pathway [26]. To date, no virus has been reports to simultaneously manipulate both PHDs-VHL and FIH. In this study, we identified that ISKNV VP077R can manipulate the HIF-pathway by simultaneously regulating VHL and FIH proteins. VP077R interacts with VHL via the HIF-binding region of VHL, competitively inhibiting the interaction between HIF-1α and VHL. Furthermore, VP077R interacts with FIH and promotes its degradation through K48-linked ubiquitination. These findings suggest that VP077R activates the HIF-pathway by simultaneously releasing two “brake proteins”, VHL and FIH. This reveals a novel mechanism by which viruses regulate the HIF-pathway (Figure 7).

Viral ANK proteins have been reported to manipulate the ubiquitination of the cellular proteins [39–41]. ORFV ANK protein 008 interacts with Skp1 to regulate SCF1 E3 complexes [39]. Ectromelia virus ANK proteins associate with Skp1 to inhibit IκBα, leading to NF-κB suppression [42]. In the present study, we found that VP077R, a viral ANK protein, interacts with Skp1. Although FIH cannot interact with Skp1, VP077R recruit FIH and Skp1 proteins to form an FIH – VP077R – Skp1 complex. These findings suggest that VP077R promotes FIH ubiquitination and degradation by recruiting these proteins. The HIF-pathway is strictly controlled by HIF hydroxylases (PHDs and FIH) [42]. Apart from directly regulating the activity of HIF hydroxylases through O_2_ availability, the abundance of HIF hydroxylases is also crucial to this pathway. The stability of PHDs is regulated by seven-in-absentia homolog RING-type E3 ubiquitin ligases [43]. However, the mechanism of FIH regulation remains unclear. Our results suggest that the stability of FIH is regulated by the SCF1 E3 complex under physiological and pathological conditions, including infectious diseases.

The “epidemiologic triad” serves as a pivotal conceptual framework for comprehending the etology of diseases [44]. For the emergence of diseases, it is imperative that the host harbours genetic susceptibility, and the pathogen exhibits genetic virulence. Furthermore, environmental factors must align favourably for disease outbreaks. Grasping how environmental conditions shape host – microbe interactions hold the key to anticipating disease outbreaks and crafting intervention strategies for disease mitigation [45]. Our preceding investigations have revealed that hypoxic environments activate the HIF-pathway, thereby augmenting the expression of functional critical viral HRE genes, notably *orf077r*, ultimately leading to heightened ISKNV replication [18]. Remarkably, in this study, we discovered that ISKNV VP077R can itself induce the HIF-pathway. Our findings suggested a complex interplay between the environment, the host, and the virus within aquatic ecosystems. Hypoxia activates the HIF-pathway, initiating a positive feedback loop between “ISKNV VP077R and the HIF-pathway” in fish harbouring ISKNV. This, in turn, triggers ISKNV replication, culminating in the onset of ISKNV disease. The intricate interplay delineated here sheds valuable light on the interconnectedness of the epidemiologic triad: virus, host, and hypoxia. Our work paves the way for the development of disease-resistant breeds, drug targets, and enhanced aquaculture models.

## Conclusions

Aquaculture provided over 17% of human consumption of animal protein and played an increasingly important role in providing food and nutrition for humans. In recent years, aquaculture towards high density, intensive to change direction. A prominent problem is the excessively high breeding density and the excessive bait feeding amount frequently led to cultured fish under hypoxia, which leads to outbreak various diseases. However, the mechanisms of how hypoxia provokes the outbreaks of infectious disease remain unclear. Herein, we found that the ISKNV encoded protein VP077R simultaneously targets VHL and FIH, thereby hijacking HIF-pathway. This finding revealed a novel mechanism of fish iridovirus could manipulate the HIF-pathway. Moreover, the stability of FIH protein was mediated by Skp1 E3 ubiquitin ligase which induces the K48-linked ubiquitination and degradation of FIH. Our work would help shed light on the mechanisms of hypoxia triggers the outbreak of iridovirus disease in intensive aquaculture.

## Data Availability

The data that support the findings of this study are openly available in figshare at https://figshare.com/, reference number [10.6084/m9.figshare.25565820].
